# Noncovalent Functionalization of Single-Walled Carbon Nanotubes with a Photocleavable Polythiophene Derivative

**DOI:** 10.3390/nano12010052

**Published:** 2021-12-25

**Authors:** Jyorthana Rajappa Muralidhar, Koichi Kodama, Takuji Hirose, Yoshihiro Ito, Masuki Kawamoto

**Affiliations:** 1 Emergent Bioengineering Materials Research Team, RIKEN Center for Emergent Matter Science, 2-1 Hirosawa, Wako 351-0198, Japan; jyorthana.rajappamuralidhar@riken.jp; 2 Graduate School of Science and Engineering, Saitama University, 255 Shimo-Okubo, Sakura-ku, Saitama 338-8570, Japan; kodama@mail.saitama-u.ac.jp (K.K.); thirose@mail.saitama-u.ac.jp (T.H.); 3 Nano Medical Engineering Laboratory, RIKEN Cluster for Pioneering Research, 2-1 Hirosawa, Wako 351-0198, Japan

**Keywords:** carbon nanotube, polythiophene, noncovalent functionalization, photocleavage, surface hydrophilicity

## Abstract

Single-walled carbon nanotubes (SWCNTs) have received extensive research attention owing to their extraordinary optical, electrical, and mechanical properties, which make them particularly attractive for application in optoelectronic devices. However, SWCNTs are insoluble in almost all solvents. Therefore, developing methods to solubilize SWCNTs is crucial for their use in solution-based processes. In this study, we developed a photocleavable polythiophene-derivative polymer dispersant for SWCNTs. The noncovalent surface functionalization of SWCNTs with a polymer allows their dispersal in tetrahydrofuran. The resultant solution-processed polymer/SWCNT composite film undergoes a hydrophobic-to-hydrophilic change in surface properties upon light irradiation (313 nm) because hydrophilic carboxyl groups are formed upon photocleavage of the hydrophobic solubilizing units in the polymer. Furthermore, the photocleaved composite film displays a 38-fold increase in electrical conductivity. This is due to the removal of the solubilizing unit, which is electrically insulating.

## 1. Introduction

Single-walled carbon nanotubes (SWCNTs) show unique electrical and optical properties [1]. Accordingly, the use of SWCNTs has led to important advances in the development of next-generation solar cells [2,3], field-effect transistors [4,5], and thermoelectric devices [6,7]. SWCNTs also show high mechanical durability owing to their extended π-conjugated lattices of sp^2^-bonded carbon atoms, yielding high-performance nanotube fibers [8].

While there are a variety of applications for SWCNTs, they also present certain drawbacks. For instance, SWCNT surfaces are hydrophobic, leading to unfavorable aggregation. Accordingly, SWCNTs are poorly dispersible and quickly precipitate in most organic solvents. Therefore, the development of new SWCNT-dispersion strategies is required for their use in solution-based processes and applications.

Noncovalent functionalization provides a means for the solution processing of SWCNTs [9,10]. When a dispersant, such as a surfactant, polymer, or biomaterial, is attached to an SWCNT surface through hydrophobic and/or π-π interactions, the uniform dispersion of the dispersant/SWCNT composite can occur without deforming its sp^2^ carbon structure [11,12]. Conjugated polymers are good candidates for use as functional dispersants [13] because extended π-structures bearing solubilizing units (e.g., alkyl chains) can bind noncovalently through π-π interactions to SWCNTs, allowing the formation of SWCNT suspensions. Subsequently, such suspensions can be used to make solution-processed SWCNT films using coating and printing techniques [14]. However, the solubilizing conjugated polymer remains attached to the surface of the SWCNTs, meaning that the resulting film can be damaged upon exposure to the solvent in which it was made. Therefore, the fabrication of insoluble SWCNT films using functional dispersants would benefit their practical application [15].

**Figure 1:**
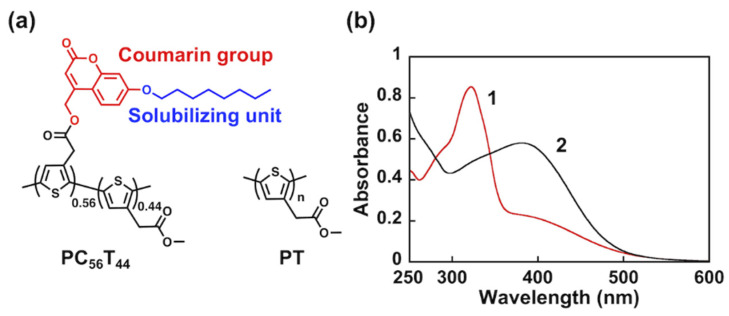
(**a**) Chemical structures of PC_56_T_44_ and PT. (**b**) Absorption spectra of (**1**) PC_56_T_44_ and (**2**) PT in THF.

In this study, we developed the polythiophene dispersant PC_56_T_44_, which features a photocleavable solubilizing unit (Figure 1a). Photoresponsive (coumarin-4-yl)methyl groups have been reported as cage compounds for biomedical applications [16,17]. We introduced an octyloxy chain as a solubilizing unit in the coumarin group. Exposure to light leads to the removal of the coumarin group, including the solubilizing unit. The novelty of this approach is that the photocleaved unit allows not only the formation of an insoluble PC_56_T_44_/SWCNT composite film, but it also changes the surface properties of the film. Upon photocleavage, the surface of the composite film changes from hydrophobic to hydrophilic. This change is due to the formation of hydrophilic carboxyl (COOH) groups on the polymer attached to the surface of the SWCNTs. Furthermore, the electrical conductivity of the photocleaved PC_56_T_44_/SWCNT composite film is 38 times that of the pristine film because the solubilizing unit is an electrical insulator.

## 2. Materials and Methods

### 2.1. Materials

Unless otherwise stated, all compounds and solvents were purchased from commercial suppliers and used without further purification. SWCNTs (≥90% carbon basis (≥80% as carbon nanotubes), 1.3 nm in diameter) were purchased from Sigma–Aldrich Co., Ltd. (St. Louis, MO, USA). Isopropanol (IPA), dichloromethane, and chloroform (CHCl_3_) were purchased from Junsei Chemical Co., Ltd. (Tokyo, Japan). Acetone, hexane, and deuterated chloroform (CDCl_3_) were purchased from FUJIFILM Wako Pure Chemical Co., Ltd. (Osaka, Japan). Tetrahydrofuran (THF; spectroscopic grade) was purchased from Kanto Chemical Co., Inc. (Tokyo, Japan). An elastic carbon-coated copper transmission electron microscopy (TEM) grid was purchased from Okenshoji Co., Ltd. (ELS-C10, Tokyo, Japan). A fused silica substrate was purchased from Matsunami Glass Ind., Ltd. (Osaka, Japan).

### 2.2. Polymer Dispersants for SWCNT

Syntheses of the polymeric dispersants PC_56_T_44_ and PT have been described elsewhere [18]. PC_56_T_44_ was afforded with a number-average molecular weight (*M*_n_) of 17,000 and a polydispersity (weight-average molecular weight (*M*_w_)/*M*_n_) of 1.4. The corresponding values for PT were *M*_n_: 6800 and *M*_w_/*M*_n_: 1.7.

The photocleavage behavior of PC_56_T_44_ was investigated by NMR and Fourier-transform infrared (FT-IR) spectroscopy. A PC_56_T_44_ film was prepared by drop-casting a CHCl_3_ solution (15 mg mL^−1^) on a fused silica substrate. The polymer film was irradiated at 313 nm (light intensity: 5 mW cm^−2^) for 5 h using a high-pressure mercury lamp (REX-250, Asahi Spectra Co., Ltd., Tokyo, Japan) equipped with a glass filter (HQBP313, Asahi Spectra). The unreacted polymer and cleaved coumarin groups were removed by immersion in CHCl_3_/hexane (1:3 (*v/v*)). After drying, the resulting polymer on the substrate was dissolved in CDCl_3_, yielding the photoirradiated NMR sample.

An FT-IR sample was prepared by drop-casting a CHCl_3_ solution (15 mg mL^−1^) onto a KBr plate. After irradiation at 313 nm for 5 h, the photoirradiated FT-IR sample was obtained by immersion in CHCl_3_/hexane (1:3 (*v/v*)) to remove the unreacted polymer and cleaved coumarin groups.

### 2.3. Dispersal of SWCNTs in THF

PC_56_T_44_ (1 mg) was dissolved in THF (10 mL) and SWCNTs (1 mg) were added to the resulting solution. The polymer/SWCNT mixture was ultrasonicated for 1 h using a tip-type ultrasonic homogenizer (Branson Sonifier 250, Branson Ultrasonics Co., Brookfield, CT, USA; power output: 20 W) in an ice bath. The suspension was centrifuged at 4000 rpm for 30 min using a Microfuge 16a centrifuge (Beckman Coulter Inc., Brea, CA, USA). A suspension of PC_56_T_44_/SWCNT (1:1 (*w/w*)) composite in THF was obtained upon collection of the supernatant layer.

A suspension of PT/SWCNT (1:1 (*w/w*)) composite was prepared in a similar way as that described for the PC_56_T_44_/SWCNT composite.

### 2.4. Preparation of PC_56_T_44_/SWCNT Composite Films

The substrates were cleaned with acetone and IPA by ultrasonication twice before preparing the samples. The substrates were treated with an ultraviolet–ozone cleaner (ASM1101N, Asumi Giken Ltd., Tokyo, Japan) for 10 min. A PC_56_T_44_/SWCNT (1:1 (*w/w*)) composite film was fabricated by drop-casting a THF solution (0.2 mg mL^−1^) onto the substrate. The obtained film was dried at 25 °C for 15 h. The PC_56_T_44_/SWCNT composite film was irradiated at 313 nm (light intensity: 5 mW cm^−2^) for 5 h. Photoirradiation was performed at 25 °C under nitrogen using a temperature controller (FP90 central processor equipped with a FP-82HT hot stage, Mettler Toledo, Columbus, OH, USA). After irradiation, the unreacted polymer and cleaved coumarin groups were removed by immersion in CHCl_3_/hexane (1:3 (*v/v*)).

### 2.5. Characterization

Absorption spectra were obtained using a V-750 (Jasco Co., Ltd., Tokyo, Japan) or UV-3600i plus spectrophotometer (Shimadzu Co., Ltd., Kyoto, Japan).

Photocleavage behavior was investigated using ^1^H NMR (JNM-ECZ400R, JEOL Ltd., Tokyo, Japan) and FT-IR (FT/IR-4100, Jasco) spectroscopies.

The SWCNT dispersion was investigated using TEM (JEM-1230, accelerating voltage: 80 kV, JEOL Ltd., Tokyo, Japan), scanning electron microscopy (SEM; Quattro ESEM, accelerating voltage: 5 kV, Thermo Fisher Scientific, Waltham, MA, USA), and Raman spectrometry (LabRAM, excitation wavelength: 633 nm, HORIBA Jobin Yvon, Kyoto, Japan).

Water contact angle measurements were obtained using a DropMaster 500 contact angle meter (Kyowa Interface Science Co., Ltd., Tokyo, Japan) equipped with a charge-coupled device camera. The water contact angle was measured immediately after 1 μL of water was dropped onto the PC_56_T_44_/SWCNT composite film with a syringe.

Film thickness was measured with a Dektak surface profiler (Bruker Co., Billerica, MA, USA).

Conductivity measurements were performed using a Keithley 2400 source meter (Tektronix Inc., Beaverton, OR, USA).

## 3. Results and Discussion

### 3.1. Photocleavage of PC_56_T_44_

Figure 1a shows the chemical structures of the polythiophene derivatives PC_56_T_44_ and PT. PC_56_T_44_ includes a (coumarin-4-yl)methyl moiety as a photocleavable group and an octyloxy side chain as a solubilizing unit. PT possesses a methyl acetate group without a photoreactive group. The absorption spectra of PC_56_T_44_ and PT in THF are shown in Figure 1b. The maximum absorption wavelength (*λ*_max_) values for PC_56_T_44_ are 322 and 381 nm. The former *λ*_max_ is due to the *S*_1_ → *S*_0_ transition of the coumarin group [19], while the latter is due to the π–π* transition of a polythiophene unit in a random coil structure [20]. Since PT also has the π–π* transition at 383 nm, PC_56_T_44_ and PT show similar π-conjugated structures in the polythiophene chain.

**Figure 2:**
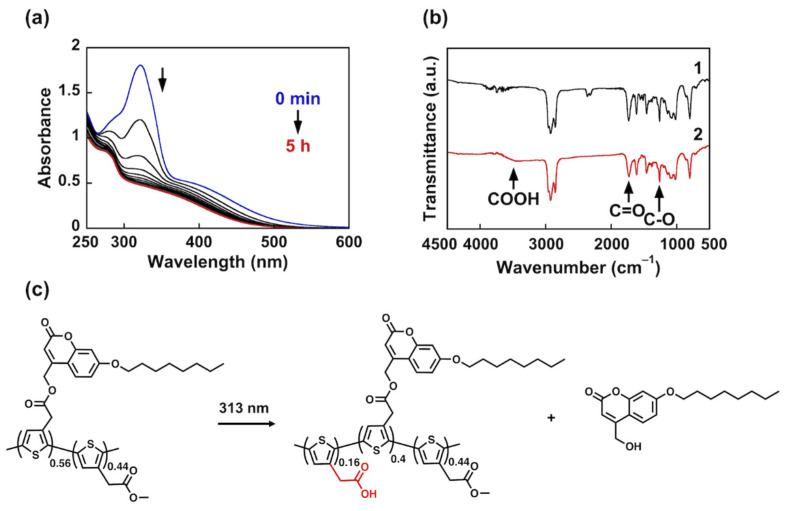
(**a**) Changes in the absorption spectra of PC_56_T_44_ upon irradiation at 313 nm in THF. (**b**) FT-IR spectra of PC_56_T_44_ (**1**) before and (**2**) after irradiation at 313 nm for 5 h. (**c**) Schematic showing the photocleavage of PC_56_T_44_.

A change in the absorption spectrum of PC_56_T_44_ in THF occurs upon photoirradiation at 313 nm (Figure 2a). As the irradiation time increases, *λ*_max_ at 322 nm decreases. After irradiation for 5 h, a photostationary state is reached. These results indicate that photoirradiation leads to photocleavage of the coumarin group bearing the solubilizing unit. As the irradiation time increases, the absorbance at 381 nm decreases. This may be due to the photocleavage of the bulky coumarin group, which changes the polythiophene conformation. Thus, the main chain shows a decrease in the conjugation length.

The FT-IR spectra of PC_56_T_44_ were obtained before and after irradiation to investigate its photocleavage behavior (Figure 2b). PC_56_T_44_ shows ester group absorbances at 1261 (C–O) and 1734 cm^−1^ (C=O) before irradiation. After irradiation, a broadened peak appeared at around 3200–3400 cm^−1^, which is attributed to a stretching mode of a carboxyl (COOH) group (Figure 2(b2)).

^1^H NMR analysis revealed that irradiation at 313 nm leads to photocleavage of the coumarin units (Figures S1 and S2). The integration value for the methylene groups in the coumarin unit (5.20 ppm) decreased from 2.46 to 1.77. Because the integration value for the methyl acetate group (3.68 ppm) did not change, 28% of the thiophene units with the coumarin groups were changed in the polymer chain. Thus, there was a 16% yield of COOH groups in the polymer chain upon photocleavage (Figure 2c). We also found that a broadening of the peak around 1730 cm^−1^ occurred in the FT-IR spectrum after irradiation (Figure 2(b2)). This may be attributed to the overlapped peaks of ester and COOH groups. Because the photocleavage rate was insufficient (16%), the peak of the COOH group around 1710 cm^−1^ could not be observed clearly.

### 3.2. Dispersion of SWCNT Using Noncovalent Functionalization

**Figure 3:**
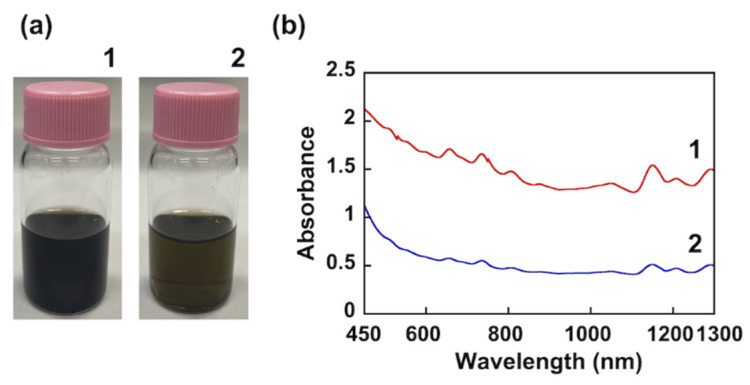
(**a**) Photographs of suspensions in THF. (**b**) Absorption spectra of suspensions in THF. (**1**) PC_56_T_44_/SWCNT composite (1:1 (*w/w*)), (**2**) PT/SWCNT composite (1:1 (*w/w*)).

Ultrasonication of polythiophene derivatives and SWCNT in THF yielded suspensions (Figure 3a). The color of the PC_56_T_44_/SWCNT composite suspension is darker than that of the PT/SWCNT composite. This indicates that PC_56_T_44_ has a better ability to disperse SWCNTs than PT.

Figure 3b shows the absorption spectra of the suspensions. The higher absorbance of the PC_56_T_44_/SWCNT composite is evidence of more dispersed SWCNTs. Thus, the π–π interactions between the aromatic coumarin in PC_56_T_44_ and the SWCNTs result in improved dispersion. The absorption spectra of the suspensions in the visible and near-infrared regions are consistent with the electronic structure of SWCNTs, corresponding to optical transitions for the first (S_11_, 940−1300 nm) and second (S_22_, 620−940 nm) semiconducting SWCNTs [21].

The broadening of an absorption peak is proportional to the degree of aggregation in a sample. Accordingly, to evaluate dispersion behavior, we measured the full width at half maximum (FWHM) at 1153 nm in the S_11_ region. The FWHM for the PC_56_T_44_/SWCNT composite was 39.4 meV. The FWHM for the poly[(9,9-dioctylfluorenyl-2,7-diyl)-*alt*-*co*-(6,6’-{2,2’-bipyridine})]/(6,5) SWCNT composite in the S_11_ region was 22.4 meV [22], indicating that our composite tended to show SWCNT aggregation in the suspension.

**Figure 4:**
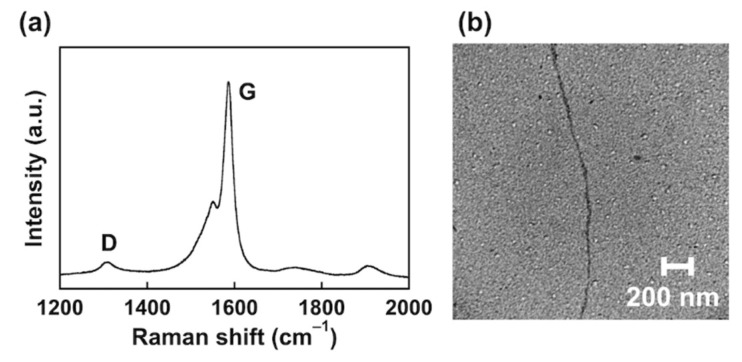
(**a**) Raman spectra of the PC_56_T_44_/SWCNT composite. Excitation wavelength: 633 nm. (**b**) TEM image of the PC_56_T_44_/SWCNT composite.

The Raman spectrum of PC_56_T_44_/SWCNT (1:1 (*w/w*)) presented two characteristic bands: the disorder (D)-band at 1317 cm^−1^ and the graphitic (G)-band at 1586 cm^−1^ (Figure 4a). The D-band is related to the presence of defects, corresponding to sp^3^-hybridized carbon in the hexagonal frameworks of the nanotubes. The G-band is attributed to the vibration of sp^2^ carbons in graphitic layers [23]. The D- and G-band intensity ratio (*I*_D_/*I*_G_) indicates the level of defects in SWCNTs. The *I*_D_/*I*_G_ value for PC_56_T_44_/SWCNT was 0.12, indicating that the SWCNTs had almost no structural defects after dispersion.

Small bundles of SWCNTs (diameter: 15−20 nm, length: 1.8 μm) were observed in the suspensions (Figure 4b). Since the SWCNT diameters were approximately 0.7−1.4 nm, the bundles of SWCNT originated from entangled structures. Since the surfaces of SWCNTs were smooth and uniform, the PC_56_T_44_ attached to the surface of SWCNTs brought about π−π interactions between the polythiophene units and the SWCNTs.

### 3.3. Morphological Change at the Surface of the Solution-Processed SWCNT Film

**Figure 5:**
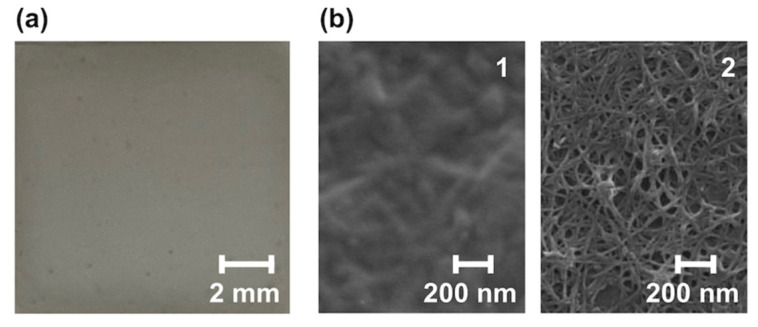
(**a**) Photograph of a PC_56_T_44_/SWCNT composite film formed on a substrate. (**b**) SEM images of the PC_56_T_44_/SWCNT composite film (**1**) before and (**2**) after irradiation at 313 nm.

We prepared a drop-cast film of the PC_56_T_44_/SWCNT composite on a fused silica substrate (Figure 5a). The film thickness was ~1.2 μm. Photocleavage of PC_56_T_44_ in the SWCNT film was investigated by SEM (Figure 5b). The as-prepared PC_56_T_44_/SWCNT composite film showed network structures. The composite bundles were 30−40 nm in diameter. However, the SEM image is not clear owing to the aggregation of SWCNTs covered with the polymer (Figure 5(b1)). In contrast, network structures were observed clearly after photoirradiation at 313 nm for 5 h. The diameters of the composite bundles decreased by 40% to 60% (Figure 5(b2)). These results suggest that the decrease in diameter was due to photocleavage of the solubilizing unit in PC_56_T_44_ on the surface of the SWCNTs. We also found that the PC_56_T_44_/SWCNT composite film was insoluble in CHCl_3_, dichloromethane, and THF after photoirradiation, owing to the photocleavage of the solubilizing unit.

**Figure 6:**
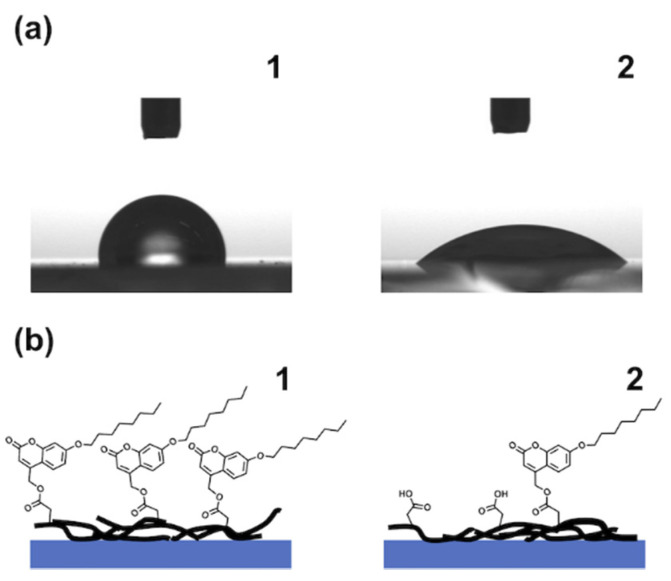
(**a**) Change in the water contact angle of the PC_56_T_44_/SWCNT composite film. (**b**) Illustration showing the photocleavage of the PC_56_T_44_/SWCNT composite film. (**1**) Before and (**2**) after irradiation at 313 nm.

Photocleavage of the solubilizing unit in PC_56_T_44_ afforded COOH groups. These groups made the SWCNT surface hydrophilic. When a surface changes from hydrophobic to hydrophilic, the water contact angle decreases. Photocleavage of the solubilizing unit decreased the water contact angle (Figure 6a). The water contact angle of the PC_56_T_44_/SWCNT composite film before irradiation was 93°. This result indicates that the surfaces of the SWCNTs were covered with PC_56_T_44_ through noncovalent functionalization. In contrast, the water contact angle after irradiation at 313 nm was 43°. Since the water contact angle for the pristine SWCNTs was around 85° [24], the change in the surface composition of the SWCNT film led to a change in the water contact angle. We also found that the formation of COOH groups upon photocleavage increased surface hydrophilicity (Figure 6b). Therefore, the composite film became hydrophilic after photocleavage of the hydrophobic solubilizing unit.

### 3.4. Modulation of the Electrical Conductivity of the PC_56_T_44_/SWCNT Composite Film upon Photocleavage

**Figure 7:**
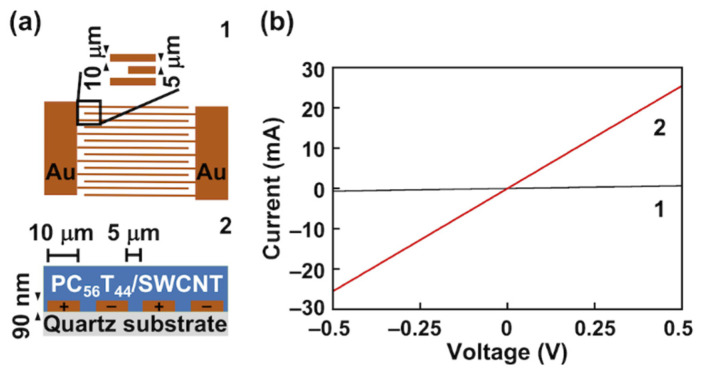
(**a**) Schematics showing the (**1**) top view of the interdigitated gold electrodes and (**2**) the side view of the sample configuration. (**b**) Current–voltage characteristics of the PC_56_T_44_/SWCNT composite film (**1**) before and (**2**) after photocleavage at 313 nm.

Finally, the electrical conductivity (*σ*) of the photocleaved composite film was investigated. Since conductive SWCNTs form network structures in films, an applied voltage generates electrical current through the nanotube junctions [25]. A PC_56_T_44_/SWCNT composite film was fabricated on interdigitated gold electrodes by drop-casting a suspension in THF (Figure 7a). Before irradiation, the PC_56_T_44_/SWCNT composite film showed a *σ* value of 2.3 × 10^−3^ S cm^−1^ (Figure 7(b1)). On the other hand, a 38-fold increase in the current was observed after photoirradiation at 313 nm (8.8 × 10^−2^ S cm^−1^; Figure 7(b2)). The *σ* value for the PC_56_T_44_/SWCNT composite was higher than that for the poly(dioctylfluorene)/SWCNT composite (4.0 × 10^−7^ S cm^−1^) [26] and the poly(3-octylthiophene)/SWCNT composite (4.7 × 10^−4^ S cm^−1^) [27]. This comparison suggests that our composite is superior for the high *σ* material. In contrast, the PT/SWCNT composite film exhibited a *σ* value of 7.7 × 10^−9^ S cm^−1^ (Figure S3). This low *σ* value may be attributed to insufficient junctions between nanotubes, owing to the poor dispersion behavior of the PT/SWCNT composite (Figure 3b).

These results suggest that the increase in *σ* was due to the photocleavage of the solubilizing unit. Because the solubilizing unit is electrically insulating, photocleavage led to a higher *σ* value for the PC_56_T_44_/SWCNT composite film. Photoinduced modulation of the *σ* value is attractive for the development of SWCNT-based film devices such as photovoltaics and thermoelectric devices.

## 4. Conclusions

We have successfully developed a photocleavable polymer dispersant PC_56_T_44_ for SWCNTs. Ultrasonication yielded dispersed PC_56_T_44_/SWCNT composite in THF through noncovalent functionalization. A solution-processed PC_56_T_44_/SWCNT composite film exhibited photocleavage of the solubilizing unit upon irradiation. Water contact angle measurements revealed that the surface properties of the composite film changed from hydrophobic to hydrophilic upon irradiation due to the formation of hydrophilic carboxyl groups. Furthermore, the photocleaved PC_56_T_44_/SWCNT composite film showed a dramatic increase in electrical conductivity because the solubilizing unit in PC_56_T_44_ acts as an electrical insulator.

A particularly important aspect of this study is the increase in electrical conductivity when the solubilizing unit was photocleaved to yield an insoluble SWCNT film. Since the photoirradiated area is insoluble, selective irradiation can form a patterned SWCNT film by solution processing. We believe that such patterned films have potential applications in printed electronics, such as photovoltaics and thermoelectric devices with p–n heterojunctions.

## Data Availability

Data is contained within the article or supplementary material.

## Supplementary Materials

The following are available online at https://www.mdpi.com/article/10.3390/nano12010052/s1: Figure S1. ^1^H NMR spectrum of PC_56_T_44_ in CDCl_3_; Figure S2. ^1^H NMR spectrum of PC_56_T_44_ after irradiation at 313 nm in CDCl_3_.; Figure S3. Current–voltage characteristics of a PT/SWCNT composite film.
