# Supplementary material for: Noncovalent Functionalization of Single-Walled Carbon Nanotubes with a Photocleavable Polythiophene Derivative
Source: Nanomaterials (Basel). 2021 Dec 25;12(1):52. doi: 10.3390/nano12010052 (PMC8746816; doi:10.3390/nano12010052)
Supplement: Supplementary file 1 [file nanomaterials-12-00052-s001.zip › nanomaterials-1483556-supplementary.pdf]

Supplementary materials

Noncovalent Functionalization of Single-Walled Carbon Nanotubes with a Photocleavable Polythiophene Derivative

Jyorthana Rajappa Muralidhar [1,2], Koichi Kodama [2], Takuji Hirose [2], Yoshihiro Ito [1,3,*] and Masuki Kawamoto [1–3,*]

[1] Emergent Bioengineering Materials Research Team, RIKEN Center for Emergent Matter Science, 2–1 Hirosawa, Wako, Saitama 351-0198, Japan; jyorthana.rajappamuralidhar@riken.jp

[2] Graduate School of Science and Engineering, Saitama University, 255 Shimo-Okubo, Sakura-ku, Saitama 338-8570, Japan; kodama@mail.saitama-u.ac.jp (K.K.); thirose@mail.saitama-u.ac.jp (T.H.)

[3] Nano Medical Engineering Laboratory, RIKEN Cluster for Pioneering Research, 2–1 Hirosawa, Wako, Saitama 351-0198, Japan

* Correspondence: y-ito@riken.jp (Y.I.); mkawamot@riken.jp (M.K.); Tel.: +81-48-467-2752 (Y.I. & M.K.); Fax: +81-48-467-9300 (Y.I. & M.K.)

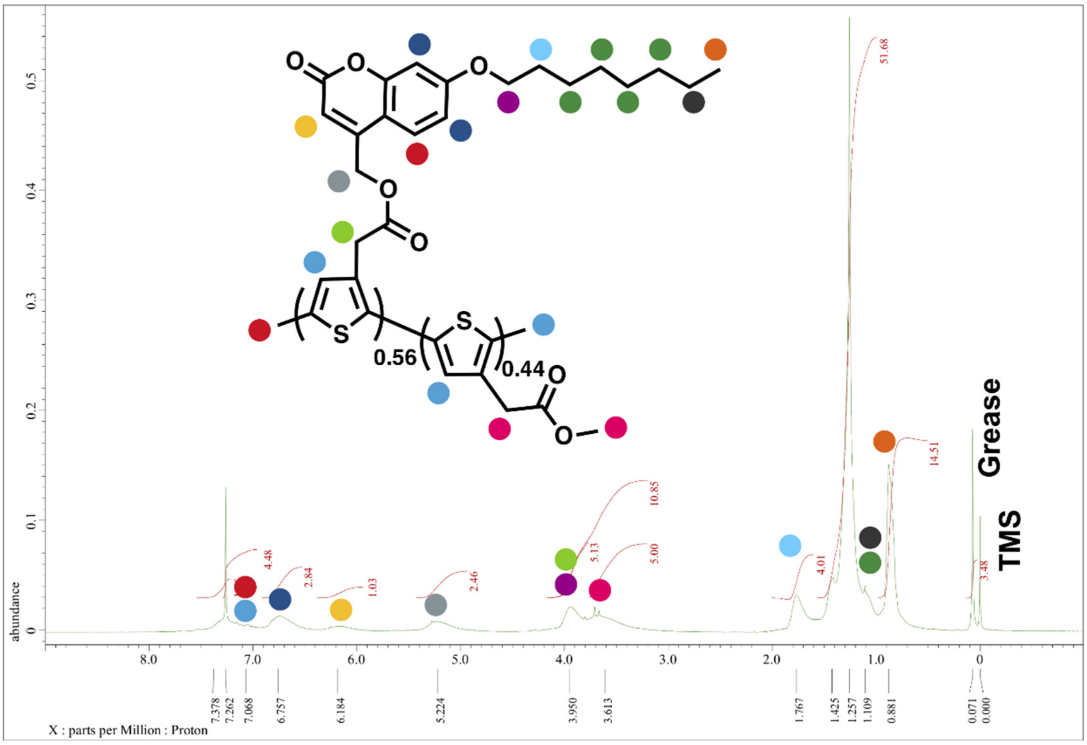

Figure S1. ^{1}H NMR spectrum of $PC_{56}T_{44}$ in $CDCl_3$.

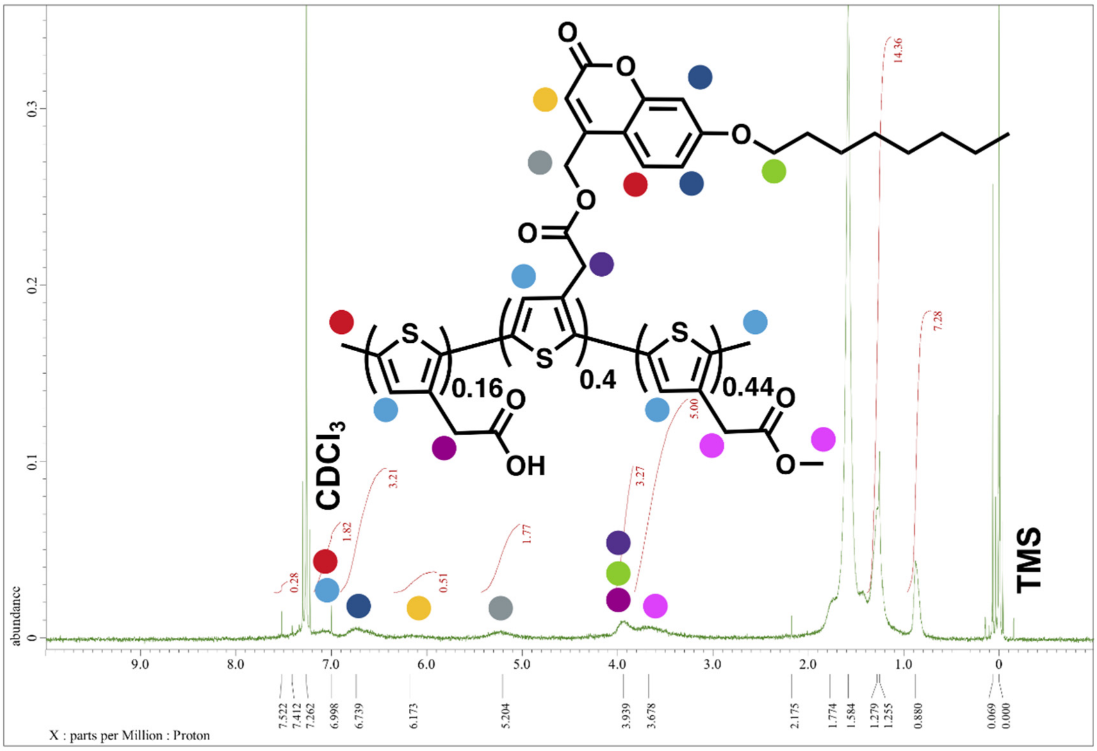

Figure S2. ^{1}H NMR spectrum of $PC_{56}T_{44}$ after irradiation at 313 nm in $CDCl_3$.

.

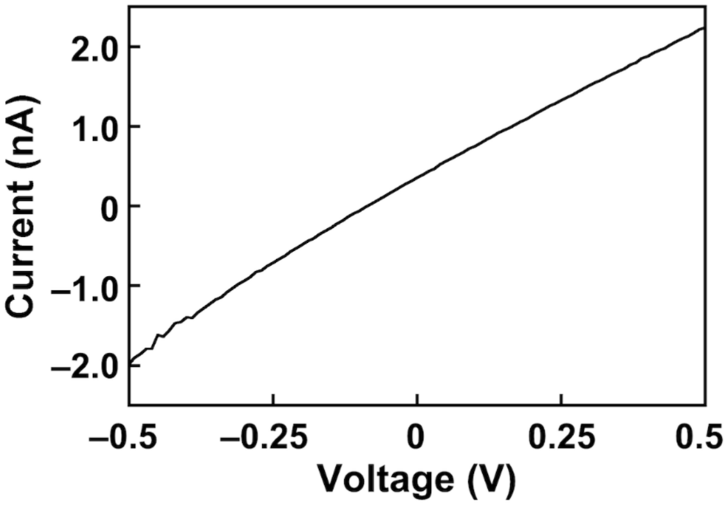

Figure S3. Current–voltage characteristics of a PT/SWCNT composite film.

Evaluation of the electrical conductivity of composite films[S1]

The total area (A_{total}) of the fingers between the electrode gaps was estimated to be

$$A_{total} = (N–1)lh$$

where N, l, and h are the number of fingers, finger length, and finger height of the electrode, respectively. σ was measured from the slope of the linear plot of the current (I) against the voltage (E), and calculated using the following equation:

$$\sigma = d\Delta I/A_{total}\Delta E$$

where d is the electrode gap.

Reference

S1. Wuelfing, W. P.; Green, S. J.; Pietron, J. J.; Cliffel, D. E.; Murray, R. W., Electronic Conductivity of Solid-State, Mixed-Valent, Monolayer-Protected Au Clusters. *J. Am. Chem. Soc.* **2000,** *122*, 11465–11472.
